# Factors associated with the risk of upper respiratory tract bacterial infections among HIV-positive patients

**DOI:** 10.1371/journal.pone.0270770

**Published:** 2022-07-07

**Authors:** Agata Skrzat-Klapaczyńska, Marcin Paciorek, Andrzej Horban, Justyna D. Kowalska

**Affiliations:** 1 Department for Adult’s Infectious Diseases, Hospital for Infectious Diseases, Medical University of Warsaw, Warsaw, Poland; 2 Hospital for Infectious Diseases, HIV Out-Patient Clinic, Warsaw, Poland; Carol Davila University of Medicine and Pharmacy: Universitatea de Medicina si Farmacie Carol Davila, ROMANIA

## Abstract

**Background:**

The risk and characteristics of upper respiratory tract (URT) bacterial infections (URT-BI) among HIV (+) patients is understudied. We analyzed factors associated with its occurrence and the spectrum of culturable pathogens among patients routinely followed at the HIV Out-Patient Clinic in Warsaw.

**Methods:**

All HIV (+) patients with available URT swab culture were included into analyses. Patients were followed from the day of registration in the clinic until first positive URT swab culture or last clinical visit from January 1, 2007 to July 31, 2016. Cox proportional hazard models were used to identify factors associated with positive URT swabs culture (those with p<0.1 in univariate included into multivariable).

**Results:**

In total 474 patients were included into the analyses, 166 with culturable URT swab. In general, 416 (87.8%) patients were male, 342 (72.1%) were infected through MSM contact, 253 (53.4%) were on antiretroviral therapy. Median follow-up time was 3.4 (1.3–5.7) years, age 35.2 (30.6–42.6) years and CD4+ count 528 (400–685) cells/μl. The most common cultured bacteria were *S*. *aureus* (40.4%) and *S*. *pyogenes* (13.9%) (Table 1). Patients with culturable URT-BI were more likely to be MSM (68.5% vs 78.9%; p<0.016), have detectable viral load (20.9% vs 12.0%; p<0.0001) and CD4+ cell count <500 cells/μl (55.2% vs 39.0%; p = 0.003) (Table 2). In multivariate survival analyses detectable viral load (HR3.13; 95%Cl: 2.34–4.19) and MSM (1.63;1.09–2.42) were increasing, but older age (0.63;0.58–0.69, per 5 years older) and higher CD4+ count (0.90;0.85–0.95, per 100 cells/μl) decreasing the risk of culturable URT-BI (Table 2).

**Conclusions:**

Culturable URT-BI are common among HIV-positive patients with high CD4+ count. Similarly to general population most common cultured bacteria were *S*. *aureus* and *S*. *pyogenes*. Risk factors identified in multivariate survival analysis indicate that younger MSM patients with detectable HIV viral load are at highest risk. In clinical practice this group of patients requires special attention.

## Introduction

The use of combination antiretroviral therapy (cART) improved the prognosis of HIV-infected patients significantly, minimizing the incidence of opportunistic diseases and significantly improving survival [1–3]. Although current cART schemes show high efficacy in inhibiting viral replication, HIV elimination is still not possible [4]. The use of cART allows partial reconstruction of the immune system, however the minimum HIV replication, even during effective therapy, is responsible for maintaining the activation of the immune system [5]. This condition is associated with an increased risk of non-AIDS-defining diseases, such as malignant tumors, cardiovascular diseases or infections [6–8]. It was observed that despite the use of cART, the frequency of deaths caused by non-AIDS-defining bacterial infections in HIV-positive population has not changed, moreover the analysis from the EuroSIDA study showed that mortality caused by non-AIDS-defining infections does not decrease with time on cART [9].

The upper respiratory tract infections (URTIs) occurs frequently in general population and they are the leading cause of acute infections in humans. URTIs are located in the upper respiratory tract and present as nasopharyngitis (common cold), sinusitis, pharyngitis, laryngitis, and laryngotracheitis [10]. Generally, they are usually considered as a self-limited disease, in 90% of cases caused by viruses. The upper respiratory tract in healthy people is colonized with many bacteria, such as *Streptococcus Pneumoniae*, *Staphylococcus aureus*, *haemophilus influenzae* or *Moraxella catarrhalis* which occasionally turn into pathogens causing infectious diseases [11]. People living with HIV (PLWH) constitute a population at high risk of developing infections due to immunodeficiency [12]. The risk and characteristics of upper respiratory tract bacterial infections (URT-BI) among PLWH is understudied. Here we analyzed factors associated with culturable URT-BI occurrence and the spectrum of culturable pathogens among patients routinely followed at the HIV Out-Patient Clinic in Warsaw.

## Material and methods

Electronic database of the HIV Out-Patient Clinic in Warsaw collects all medical information on patients routinely followed since 1994. The study included patients who reported to the clinic after January 1, 2007, due to the prospective management of the electronic database from that date, to July 31, 2016. All PLWH with available URT swab culture were included into analyses. Patients were followed from the day of registration in the clinic until first positive URT swab culture or last clinical visit.

Inclusion criteria for the study consisted of the date of the first visit to the clinic after January 1, 2007; age over 18; performing at least one culture from the upper respiratory tract during the study period. Exclusion criteria from the study included patients who did not have a single CD4 count and / or HIV RNA test; patients who were cultured on the day of the first clinic registration—due to the lack of observation time required for survival analysis; patients who had a CD4 count performed earlier than 180 days prior to the event; patients with HIV viral load performed earlier than 360 days prior to the event.

The total number of patients attending the clinic during the study timespan was 4428. Of these, 3,348 patients had never been cultured and were excluded from the analysis. 559 was the total number of patients for which a swab culture was requested and among these 474 was the total number of patients with evaluable records included in the study. Finally, 166 patients had positive swab culture and 308 patients had negative swab culture.

Our study identified pathogens as cultured bacteria. For this reason, the term culturable URT-BI should be adopted as the result of our microbiological research.

For the detection of bacteria from the upper respiratory tract the following culture substrates were used: lamb blood agar and Schaedler’s medium as well as isolation culture media: Chapman’s substrate and MacConkey substrate. For HIV RNA identification Abbott RealTime HIV-1 Test was used. For the determination of CD4/CD8, blood samples were collected by venipuncture directly into a sterile BD Vacutainer® EDTA (ethylenediaminetetraacetic acid) tube. Identification, determination of percentages and absolute numbers of mature human T (CD3 +), helper / inducer (CD3 + CD4 +) T lymphocytes and suppressor / cytotoxic (CD3 + CD8 +) T lymphocytes in whole blood were performed using a single test tubes—BD Tritest—tri-color direct immunofluorescent reagent. Antibodies were stained with: CD4 fluorescein chlorocellulose (FITC) / CD8 phycoerythrin (PE) / CD3 peridine (PerCP).

In statistical analyses non-parametric tests were used for group comparison as appropriate. Kaplan-Meyer curves were used to investigate the time to first URT-BI in those who reported to clinic after January 1, 2007. The follow-uptime was calculated from the day of registration in the clinic until first positive URT swab culture or last clinical visit.

Univariate and multivariate Cox-proportional hazard models were used to identify factors which were associated with higher chance URT-BI. Factors considered for inclusion were age, gender, last CD4+, last CD8+, HIV RNA, antiretroviral treatment, route of infection. All analyses were performed using Statistical Analysis Software Version 9.3 (Statistical Analysis Software, SAS Institute North Carolina USA version 9.4,).

## Ethical approvals

The study was approved by the Bioethical Committee of the Medical University of Warsaw (Nr AUBE/102/15). The waiver for informed consent was given due to the retrospective nature of the study.

## Results

In total 474 patients were included into the analyses. In general, 416 (87.8%) patients were males and 58 (12,2%) patients were females. In total, 342 (72.1%) patients were infected through MSM contact, 88 (18,6%) through heterosexual contact. Among the studied population 253(53.4%) patients were on antiretroviral therapy, 338 (71,3%) patients had undetectable viral load. Median follow-up time was 3.4 (1.3–5.7) years, age 35.2 (30.6–42.6) years and CD4+ count 528 (400–685) cells/μl (Table 2).

One hundred and sixty six patients had positive URT swab culture. The most common cultured bacteria were *S*. *aureus* (40.4%) and *S*. *pyogenes* (13.9%) (Table 1).

**Table 1:** Cultured bacteria identified in upper respiratory tract swab cultures.

Cultured bacteria	N	%
Total number	166	100
*Staphylococcus aureus*	67	40,4
*Streptococcus pyogenes*	23	13,9
*Streptococcus group C*, *F*, *G*	15	9,02
*Haemophilus influenzae*	11	6,6
*Klebsiella pneumoniae*	10	6
2 types of bacteria	10	6
*Streptococcus pneumoniae*	9	5,4
*Haemophilus parainfluenzae*	5	3,6
*Enterobacter cloace*	2	1,2
*Escherichia coli*	2	1,2
3 types of bacteria	1	0,6
*Citrobacter spp*	1	0,6
*Enterobacter aerogenes*	1	0,6
*Enterococcus agglomerens*	1	0,6
*Moraxella catarhalis*	1	0,6
*Proteus mirabilis*	1	0,6
*Serratia marscescens*	1	0,6
*Staphylococcus epidermidis*	1	0,6
*Staphylococcus haemolyticus*	1	0,6
*Streptococcus agalactiae*	1	0,6
*Streptococcus viridans*	1	0,6

Patients with culturable URT-BI were more likely to be MSM (68.5% vs 78.9%; p<0.016), have detectable viral load (20.9% vs 12.0%; p<0.0001) and CD4+ cell count <500 cells/μl (55.2% vs 39.0%; p = 0.003) (Table 2).

**Table 2:** Baseline characteristics for the groups of patients with positive and negative upper respiratory tract culture.

Characteristic Total N = 474	N total	Positive upper respiratory tract culture N = 166	Negative upper respiratory tract culture N = 308	P value
	N(%)			
Female	58 (12.2)	13 (7.8)	45 (14.6)	0.03^a^
Male	416 (87.8)	153 (92.2)	263 (85.4)	
Risk group				0.01^a^
Heterosexual	88(18.6))	18 (10.8)	70 (22.7)	
MSM	342(72.1)	131 (78.9)	211(68.5)	
IDU	33 (6.9)	12 (7.2)	21(6.8)	
Unknown	11 (2.3)	5 (3)	6 (1.9)	
ARV	221 (46.6)	78(47)	143 (46.4)	
No ARV	253 (53.4)	88(53)	165(53.6)	0.90^a^
Detectable viral load	136 (28.7)	99 (59.6)	37 (12)	<0.0001^a^
Undetectable viral load	338 (71.3)	67 (40.4)	271 (88)	
Baseline CD4				0.003^a^
<350 cells/ul	75 (15.9)	31 (18.8)	44(14.3)	
350–500 cells/ul	136 (28.7)	60 (36.4)	76 (24.7)	
>500 cells/ul	262 (55.4)	74 (44.8)	188 (61)	
	Median (IQR)			
Age at registration in years, median [IQR*]	35.2 (30.6–41.6)	31.6 (26.7–36.9)	37.1 (32.5–44.5)	0.001^b^
In care from 2007 in years, median [IQR*]	3.43 (1.34–5.74)	0.70 (0.21–1.84)	4.66 (3.30–7.00)	0.189^b^
Baseline CD4 in cells/ul, median [IQR*]	528 (400–685)	483 (374–604)	559 (435–716)	<0.0001^b^
Baseline CD8 in cells/ul, median [IQR*]	851 (628–1122)	941(674–1274)	809(600–1051)	0.03^b^

*IQR—interquartile range.

^a^Chi2 test.

^b^ Kruskal-Wallis test.

**Fig 1:**
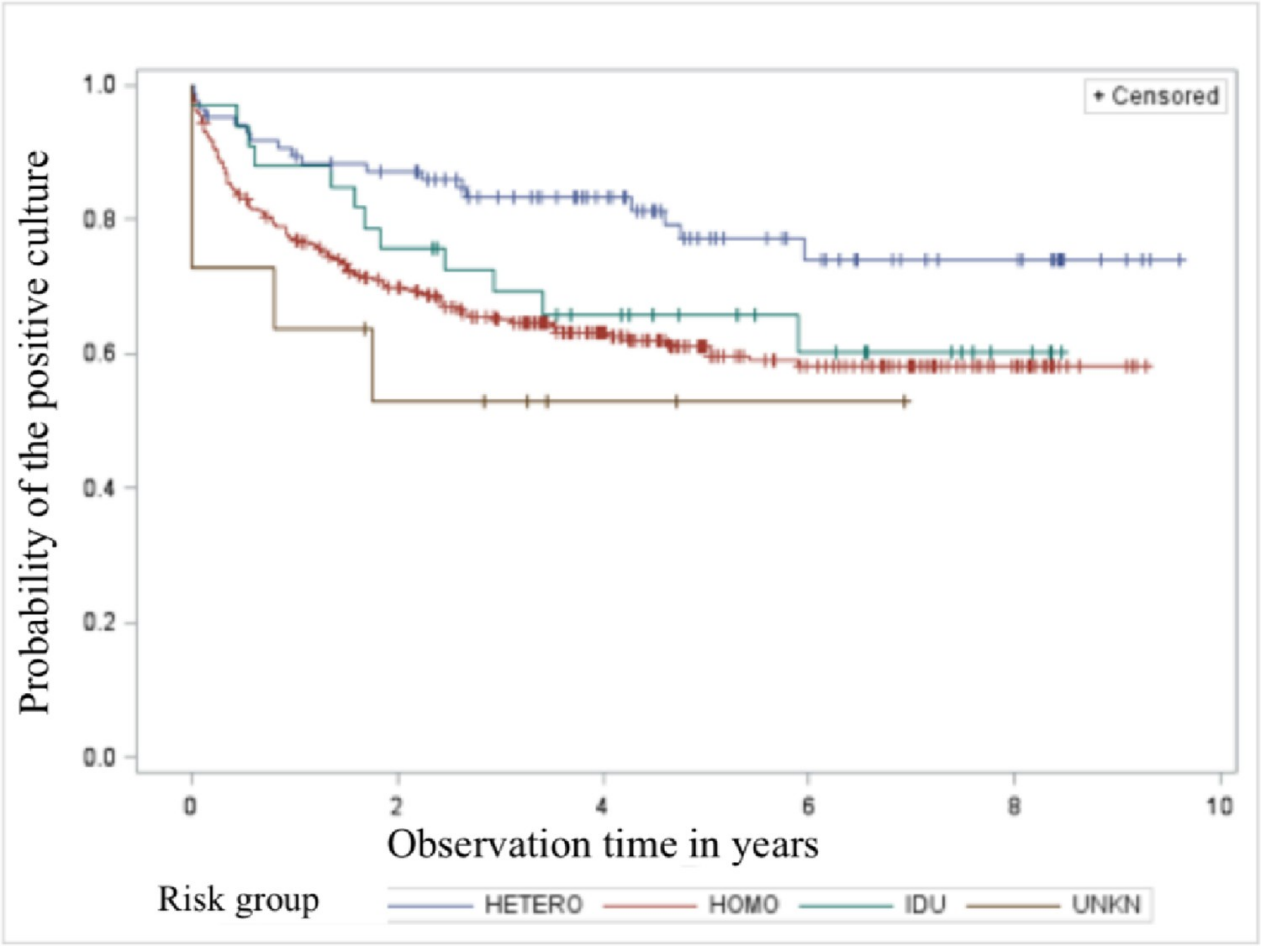
Probability of a positive culture result according to Kaplan Meier with stratification relative to the route of infection (Log-Rank p = 0.0137).

Probability of a positive URT culture according to Kaplan Meier with stratification relative to the route of infection (Log-Rank p = 0.0137) was higher in patients infected through homosexual contact and among patients, who have never admitted what was the route of HIV infection (Fig 1).

In univariate survival analyses factors identified as significant were age (HR 0.901; 95% Cl: 0.879–0.924 per 5 years older), last CD4+ (0.999; 0.998–0.999; per 100 cells/μl), last CD8+ (1.001; 1.000–1.001; per 100 cells/μl) and detectable viral load (6.366; 4.640–8.735; >50 copies/ml) In multivariate survival analyses detectable viral load (HR 3.132; 95% Cl 2.342–4.189; >50 copies/ml) and MSM (1.633; 1.098–2.428) were increasing the risk of culturable URT-BI; whereas older age (0.633; 0.897–0.929, per 5 years older) and higher CD4+ count (0.999; 0.998–1.000, per 100 cells/μl) were decreasing the risk of culturable URT-BI (Table 3).

**Table 3:** Univariate and multivariate survival analyses for positive upper respiratory tract swab culture.

		Univariate		Multivariate*	
Variable		HR (95% CI)	P value	HR (95% CI)	P value
Age in years (per 5 years)		0.901 (0.879–0.924)	<0.0001^b^	0.913 (0.897–0.929)	<0.0001^b^
Last CD4+ cells/ul (per 100)		0.999 (0.998–0.999)	<0.0001^b^	0.999 (0.998–1.000)	0.0005^b^
Last CD8+ cells/ul (per 100)		1.001 (1.000–1.001)	<0.0001^b^	1.000 (1.000–1.000)	0.12^b^
HIV RNA >50 copies/ml		6.366 (4.640–8.735)	<0.0001^a^	3.132 (2.342–4.189)	<0.0001^a^
Route of infection heterosexual	MSM	2.105 (1.285–3.450)	0.003^a^	1.633 (1.098–2.428)	0.01^a^
	IDU	1.766 (0.851–3.667)	0.12^a^	0.903 (0.571–1.428)	0.66^a^
	Unknown	3.236 (1.201–8.722)	0.02^a^	2.274 (0.995–5.200)	0.005^a^
Male gender		1.828 (1.037–3.223)	0.03^a^	-	-
On antiretroviral treatment		0.900 (0.661–1.225)	0,50^a^	-	-

* Adjusted for all variables significant (p<0.1) in univariable analyses.

^a^Chi2 test.

^b^ Kruskal-Wallis test.

## Discussion

In our study one in three observed patients had positive URT culture and the most common cultured bacteria was *S*. *aureus* (40,4%), followed by *S*. *pyogenes* (13.9%).

In the general population, bacteria responsible for acute pharyngitis and tonsillitis in 5–10% is *Streptococcus pyogenes* in adults, while *group C* and *G streptococci* are found in 5–11% of cases [13,14]. Bacterial acute rhinosinusitis in the general population is caused in most cases by *S*. *pneumoniae* (26–35%) and *H*. *influenzae* (21–40%). The remaining microbes are responsible for less than 20% of bacterial infections and include *M*. *catarrhalis*, *S*. *aureus* and *S*. *pyogenes* [15,16].

In our findings *Haemophilus influenzae* and *Streptococcus pneumoniae* were quite common bacteria– 6,6% and 5,5% respectively but *S*.*aureus* was the most common cultured bacteria.

*Staphylococcus aureus* is isolated from healthy people from the anterior throat, digestive tract, urogenital tract and skin. About 30% of healthy people carry *S*. *aureus* in the nasopharynx, and the transmissible carrier found in about 50–60% of the general population. Increased carriage was found in patients with type 1 diabetes, hemodialyzed patients, patients with peritoneal dialysis, intravenous drug users, and HIV-infected patients [17,18]. The life-threatening infections of *S*. *aureus* are frequently descended from commensal bacteria colonizing the nose [19]. Asymptomatic colonization of *S*.*aureus* can lead to common bacterial infection or more severe illnesses, especially in the specific populations. URT colonization is an important risk factor for staphylococcal septicemia and colonized subjects develop infection more frequently compared to non-carriers [19]. In PLWH, *S*.*aureus* infection account for significant morbidity [20]. For this reason, cultured S. aureus strains from the upper respiratory tract may only be colonizers, but they can also become a serious URT-BI pathogens.

In 2009, a study by Shet et al. was published, which compared the colonization and subsequent infection with the etiology of methicyllin-resistant Staphylococcus aureus (MRSA) among 107 people infected with HIV compared to the group of 52 people without HIV infection. Analysis of the total colonization frequency by *S*. *aureus* showed that 58.9% HIV infected and 34.6% of non-HIV infected subjects were colonized at least once during the study period. During the study, ten people infected with HIV developed a skin and soft tissue infection with the etiology of MRSA, and none of the non-HIV positive was described during the same period. All persons who had an MRSA infection were also carriers of MRSA [18]. In the conducted research, it was proved that colonization of the skin and mucous membranes with MRSA is a predisposing factor for a full-blown infection with this pathogen [21]. This trend was also observed among HIV infected populations with an increased incidence of both colonization and infection [22,23].

Patients infected with human immunodeficiency virus are particularly susceptible to MRSA infections because they have reduced immunity as well as due to demographic, behavioral and socio-economic factors as well as frequent exposures to the healthcare system [24,25]. In PLWH, MRSA ranks high among the most common causes of bacterial infections, especially skin and soft tissue infections [22]. The new scientific reports more and more often include information that the colonization of Staphylococcus aureus may have an impact on the more frequent incidence of respiratory tract infections of this etiology in patients with immunodeficiency, e.g. hospitalized in ICU and mechanically ventilated [26].

In our study in multivariate survival analyses detectable viral load was increasing the risk of culturable URT-BI by more than 3 times.

Reekie et al. in 2011 showed that HIV infected individuals with CD4 + cell counts greater than 350 cells / μl and those with uncontrolled viral replication had an increased incidence of AIDS-related diseases and a slightly increased incidence of non-AIDS defining diseases. The relationship with AIDS was clear and consistent. However, after considering the complicating factors, there was also a relationship with the occurrence of non-AIDS-related events, but no differences were observed between mean and high viral load [27]. In the study from Sogaard et al. performed in 2013 there was clearly demonstrated that discontinued antiretroviral therapy is a risk factor for bacterial infections (IRR = 2.96, 95% CI: 2.03–4.32) in HIV-infected patients [28]. It is now known that undetectable HIV viral load is achieved by uninterrupted ARV treatment, and non-recommended interruptions in ARV treatment result in detectable HIV viral load and its consequences [29]. These data are in line with the results of our study which found an association between HIV viral load> 50 copies / ml and a more than 3-fold increased risk of positive cultured bacteria.

More work has been done for serious bacterial infections in HIV-positive patients, but we can observe trends in occurrence of these events in the combination ART era. For example, the incidence of pneumonia and other opportunistic infections has decreased [30,31]. Observational studies have shown that the introduction of cART led to a decrease in the frequency of hospitalizations associated with pneumonia, but the risk of pneumonia remained still six-fold higher among people with HIV than people without HIV. Similar trends are also observed in the case of bacterial meningitis and invasive pneumococcal disease [28,32]. However, bacterial pneumonia and chronic obstructive pulmonary disease (COPD) are the most prevalent lung comorbidities in people living with HIV [33]. Moreover, subsequent studies have suggested that COPD, lung cancer and pulmonary hypertension may also occur with greater frequency in populations infected with HIV [34–36]. Regarding URT-BI among PLWH, we still find the gap in the studies and the factors associated with its occurrence and the spectrum of pathogens are not well researched.

In our multivariate survival analyses older age was decreasing the risk of culturable URT-BI. This is completely the opposite situation considering the lower respiratory tract infections where the factor increasing the risk of these infections is older age [37]. Our results may be related to a typical epidemic trend, because young people usually stay in groups.

There are some limitations in our study which need to be mentioned. First of all, data on clinical symptoms and indications for URT swab culture were not available for these analyses. This could result in underreporting of bacterial infections of the URT. However, these data represent clinical practice therefore it is not unreasonable to assume that clinical symptoms were the indications for performing a swab. Secondly, it was the fact that the role of *Staphylococcus aureus* as the pathogen of the upper respiratory tract is the subject of discussion because it is a frequent component of the flora found in the nasal tracts in health. In the shared database, it was not possible to verify disease symptoms with a positive culture result. Moreover, we didn’t know if our *Staphylococcus aureus* was MSSA or MRSA. Therefore, our findings can only be referred to population of HIV-positive patients who had URT swab culture-based diagnosis.

Infections remain a leading cause of global mortality, with bacterial pathogens among the greatest concern [38]. Among PLWH, research works usually focus on serious bacterial infections, but mild bacterial infections, such as URT-BI, which can lead to serious complications, are understudied. In our opinion the analysis of factors affecting the occurrence of bacterial upper respiratory tract infections in PLWH, with particular emphasis on antiretroviral treatment, remains a research challenge. Thanks to combination ARV, we can observe the aging of this population and better quality of life, but it is important to investigate whether URT-BI are more frequent than in the general population. Due to the fact that the detectable viral load of HIV increases the risk of upper respiratory tract bacterial infection, we should realize that the benefits of effective antiretroviral therapy also include reduction of URT-BI events.

## Supporting information

S1 Data (XLS)

S2 Data (XLSX)
